# Mindfulness-based stress reduction for low back pain. A systematic review

**DOI:** 10.1186/1472-6882-12-162

**Published:** 2012-09-25

**Authors:** Holger Cramer, Heidemarie Haller, Romy Lauche, Gustav Dobos

**Affiliations:** 1 Chair of Complementary and Integrative Medicine, University of Duisburg-Essen, Essen, Germany

**Keywords:** Low back pain, Mindfulness-based stress reduction, MBSR, Complementary therapies, Review

## Abstract

**Background:**

Mindfulness-based stress reduction (MBSR) is frequently used for pain conditions. While systematic reviews on MBSR for chronic pain have been conducted, there are no reviews for specific pain conditions. Therefore a systematic review of the effectiveness of MBSR in low back pain was performed.

**Methods:**

MEDLINE, the Cochrane Library, EMBASE, CAMBASE, and PsycInfo were screened through November 2011. The search strategy combined keywords for MBSR with keywords for low back pain. Randomized controlled trials (RCTs) comparing MBSR to control conditions in patients with low back pain were included. Two authors independently assessed risk of bias using the Cochrane risk of bias tool. Clinical importance of group differences was assessed for the main outcome measures pain intensity and back-specific disability.

**Results:**

Three RCTs with a total of 117 chronic low back pain patients were included. One RCT on failed back surgery syndrome reported significant and clinically important short-term improvements in pain intensity and disability for MBSR compared to no treatment. Two RCTs on older adults (age ≥ 65 years) with chronic specific or non-specific low back pain reported no short-term or long-term improvements in pain or disability for MBSR compared to no treatment or health education. Two RCTs reported larger short-term improvements of pain acceptance for MBSR compared to no treatment.

**Conclusion:**

This review found inconclusive evidence of effectiveness of MBSR in improving pain intensity or disability in chronic low back pain patients. However, there is limited evidence that MBSR can improve pain acceptance. Further RCTs with larger sample sizes, adequate control interventions, and longer follow-ups are needed before firm conclusions can be drawn.

## Background

Low back pain is a major public health problem, with 76 % of the population experiencing low back pain in a given year [1]. It has become the largest category of medical claims, placing a major burden on individuals and health care systems [2]. Low back pain is the most common condition for which complementary therapies are used [3]. In the US, more than half of patients suffering from low back pain use complementary therapies [4].

Mindfulness is the common ground of several complementary therapies. Derived from Buddhist spiritual tradition, mindfulness has been secularized and integrated into behavioral treatment approaches [5]. While mindfulness has been described as the core construct of Buddhist meditation [5], it also comprises a specific state of consciousness that has been characterized as non-elaborative, non-judgmental moment-to moment awareness, a way to accept and trust in one’s own experience [6]. Therefore, mindfulness-based therapies not only include training in so-called formal practice of mindfulness, this is meditation, but also training in so-called informal practice of mindfulness, this is retaining a mindful state of consciousness during routine activities in everyday life [7,8].

The most commonly used mindfulness-based intervention is mindfulness-based stress reduction (MBSR). MBSR has originally been developed in a behavioral medicine setting for patients with chronic pain and stress-related complaints [9,10]. MBSR is a structured 8-week group program of weekly 2.5-hour sessions and 1 all-day (7 to 8-hour) silent retreat. Key components of the program are sitting meditation, walking meditation, hatha yoga and body scan, a sustained mindfulness practice in which attention is sequentially focused on different parts of the body [6]. Another important component is the transition of mindfulness into everyday life.

Mindfulness-based cognitive therapy (MBCT) combines MBSR with cognitive-behavioral techniques [11,12]. It retains the original 8-week group-based approach. Originally developed as a treatment for major depression [11], MBCT is more and more adapted for other specific conditions [12]. Other mindfulness-based interventions include mindful exercise [13] and acceptance and commitment therapy [14] that do not necessarily include formal meditation practice.

Pain has been a key topic of research on MBSR from the beginning [9]. Several trials assessed the effect of MBSR on patients with heterogeneous chronic pain conditions, mainly reporting positive results [15-19]. A recent comprehensive meta-analysis of mindfulness-based interventions for chronic pain conditions found small effects on pain, depression and physical well-being when considering only randomized controlled trials [14]. However, this meta-analysis included only one trial on low back pain.

The aim of this review was to systematically assess and - if possible - meta-analyze the effectiveness of MBSR and MBCT in patients with low back pain.

## Methods

PRISMA guidelines for systematic reviews and meta-analyses [20] and the recommendations of the Cochrane Collaboration [21] were followed.

### Literature search

The literature search comprised the following electronical databases from their inception through November 2011: Medline, EMBASE, the Cochrane Library, PsycINFO, and CAMBASE. The complete search strategy for Medline was as follows: (MBSR[Title/Abstract] OR MBCT[Title/Abstract] OR mindful*[Title/Abstract]) AND (low back pain[MeSH Terms] OR low back pain[Title/Abstract] OR lower back pain[Title/Abstract] OR lumbago[Title/Abstract] OR low backache[Title/Abstract] OR low back ache[Title/Abstract] OR sciatica[MeSH Terms] OR sciatica[Title/Abstract]). The search strategy was adapted for each database as necessary. No language restrictions were applied. In addition, reference lists of identified original articles were searched manually. All retrieved articles were read in full to determine eligibility.

### Eligibility criteria

#### Intervention

Studies that assessed MBSR or MBCT as the main intervention were included. Studies on mindfulness-based interventions that were clearly different from the original MBSR/MBCT programs, such as mindful exercise or acceptance and commitment therapy, were excluded while studies that used variations of the MBSR/MBCT programs, such as variations in program length, frequency or duration were included.

#### Study type

Only randomized controlled trials (RCTs) were included, while observational studies or non-randomized trials were excluded. No treatment (“wait-list”), usual care or any active treatment were acceptable as control interventions.

Studies were included only if they were published as full-text articles in peer reviewed scientific journals.

#### Patients

Studies of patients with a diagnosis of low back pain were included regardless of pain cause, duration and intensity.

### Data extraction

Two reviewers independently extracted data on characteristics of the study (e.g. trial design, randomization, blinding), characteristics of the patient population (e.g. sample size, age, diagnosis), characteristics of the intervention and control condition (e.g. type, program length, frequency and duration), drop-outs, outcome measures, follow-ups, results and safety. Discrepancies were rechecked with a third reviewer and consensus achieved by discussion.

#### Risk of bias in individual studies

Risk of bias was assessed by two authors independently using the Cochrane risk of bias tool. This tool assesses risk of bias on the following domains: selection bias, performance bias, detection bias, attrition bias, reporting bias, and other bias [21]. Discrepancies were rechecked with a third reviewer and consensus achieved by discussion. Trial authors were contacted for further details if necessary.

### Data analysis

Main outcome measures were pain intensity and back-related disability. Safety was defined as secondary outcome measure. Other outcome measures used in the included studies were analyzed exploratively.

Meta-analysis was planned if sufficient homogeneous RCTs were available for statistical pooling. However, as only 3 RCTs were available that were heterogeneous regarding characteristics of patients, interventions, and control conditions, no meta-analysis was performed.

To determine clinical importance of group differences the following criteria were used: 10 mm (or 10 %) difference in post-treatment scores or change scores on a 100 mm visual analog scale of pain intensity [22], and 2–3 points (or 8 %) difference in post-treatment or change scores on the Roland-Morris Disability Questionnaire for back-specific disability [23].

## Results

### Literature search

**Figure 1:**
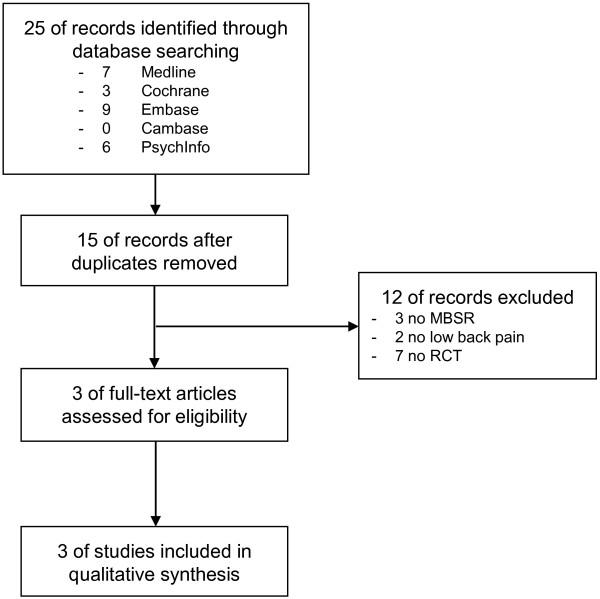
Flowchart of the results of the literature search.

Twenty-five records were retrieved in literature search, 10 of them were duplicates. Three full-text articles with a total of 117 patients were assessed for eligibility and all of them were eligible for qualitative analysis (Figure 1).

### Study characteristics

Characteristics of the study, patient population, intervention, control condition, outcome measures, follow-ups and results are shown in Table 1.

**Table 1:** Characteristics of the included studies

**Author, year**	**No. of participants, No. of groups**	**Mean age**	**Inclusion criteria**	**Treatment group: Intervention Program length, frequency, duration**	**Control group: Intervention Program length, frequency, duration**	**Longest follow-up**	**Outcome measures**	**Results**^a^
								**a) at post treatment**
								**b) at follow up**
Esmer et al. 2010 [24]	40, 2	55.2 ± 11.2 (MBSR); 54.9 ± 9.5 (CG)	Persistent leg pain, low back pain or both, Lumbosacral spinal surgery within the last 2 years	MBSR according to the curriculum developed at the University of Massachusetts 8-week program, once weekly for 1.5 to 2.5 hours plus one 6-hour session Including gentle yoga Homework: 45 min. meditation each day Additional usual medical care allowed	Waiting list control group Additional usual medical care allowed	40 weeks (in MBSR only)	1. Pain Acceptance (CPAQ)	1a. MBSR > CG, p = 0.014
							2. Disability (RMDQ)	2a. MBSR > CG, p = 0.005
							3. Pain Intensity (VAS)	3a. MBSR > CG, p = 0.021
							4. Sleep Quality (Abridged PSQI)	4a. MBSR > CG, p = 0.047
							5. Analgesic Medication Log	5a. MBSR > CG, p = 0.001
								1b-5b. All effects were maintained within MBSR
Morone et al. 2008 [25]	37, 2	74.1 ± 6.1 (MBSR); 75.6 ± 5.0 (CG)	65 years of age or older, MMSE ≥ 23, Chronic low back pain (with moderate intensity for at least 3 months)	MBSR according to the curriculum developed at the University of Massachusetts Without yoga 8-week course, once weekly for 1.5 hours Homework: 45 min. meditation each day	Waiting list control group	3 months (in MBSR only)	1. Pain Intensity (MPQ-SF, SF-36 Pain Scale)	1a. NS
							2. Pain Acceptance (CPAQ)	2a. Total Score: MBSR > CG, p = 0.008; Activities Engagement: MBSR > CG, p = 0.004
							3. Quality of Life (SF-36)	3a. NS
							4. Disability (RMDQ, SPPB, SF-36 Physical Functioning Scale)	4a. SF-36 Physical Functioning Scale: MBSR > CG p = 0.03; NS for RMDQ and SPPB
								1b-4b. NS when compared to post treatment assessment
Morone et al. 2009 [26]	40, 2	78.0 ± 7.1 (MBSR); 73.0 ± 6.2 (CG)	65 years of age or older, MMSE ≥ 24, Chronic low back pain (with moderate intensity for at least 3 months)	MBSR according to the curriculum developed at the University of Massachusetts Without yoga 8-week course, once weekly for 1.5 hours Homework: 45 min. meditation each day	Health education program 8-week course, once weekly for 1.5 hours Homework: mental exercise each day	4 months	1. Disability (RMDQ)	1a. NS
							2. Pain Intensity (MPQ-SF, SF-36 Pain Scale)	2a. NS
							3. Self-efficacy (CPSS)	3a. NS
							4. Quality of Life (SF-36 Role Emotional Scale)	4a. MBSR > CG, p < 0.05
							5. Mindfulness (MAAS, FFMQ)	5a. NS
								1b-5b. NS

Abbreviations: CG – control group; CPAQ – Chronic Pain Acceptance Questionnaire; CPSS – Chronic Pain Self-Efficacy Scale; FFMQ – Five Facet Mindfulness Questionnaire; MAAS – Mindful Attention Awareness Scale; MBSR – Mindfulness-Based Stress Reduction; MMSE – Mini-Mental Status Exam; MPQ-SF – McGill Pain Questionnaire Short Form; NS – not significant; PSQI – Pittsburgh Sleep Quality Index; RMDQ – Roland Morris Disability Questionnaire; SF-36 – Medical Outcomes Study 36-item short-form survey; SPPB – Short Physical Performance Battery; VAS – Visual Analog Scale.

^a^ > indicates “significantly better than”.

#### Setting and patient characteristics

All 3 included RCTs were conducted in the USA. Patients were recruited from a multidisciplinary spine and rehabilitation center [24], an adult pain clinic [25], and by posted flyers and newspaper advertisements [25,26]. Patients in 2 RCTs were older adults (age ≥ 65 years) with chronic (duration ≥ 3 months) low back pain [25,26]. In one of the two RCTs, minimal pain intensity was not defined [25] while in the other RCT pain had to be of at least moderate intensity on the “pain thermometer” [26]. Patients with non-specific low back pain, as well as specific low back pain, mainly due to osteoarthritis, were included [25,26]. The third RCT included patients of any age with failed back surgery syndrome; this is persistent back pain and/or leg pain of any duration and any intensity that persisted after lumbosacral surgery (within ≤ 2 years) [24].

#### MBSR

All included RCTs used MBSR interventions that were adapted from the original MBSR program developed at the University of Massachusetts. The two trials of older adults [25,26] utilized adapted 8-week programs with weekly 90-minute sessions. Roughly half of each session was dedicated to mindful meditation (body scan, sitting meditation, walking meditation), the other half to education and discussion. The programs did not incorporate yoga or an all-day silent retreat.

Patients in the trial on failed back surgery syndrome [24] participated in a MBSR intervention including 8 weekly 2.5 to 3.5-hour sessions and an additional 6-hour session in the 6^th^ week. Besides education, the program included mindful meditation (sitting meditation, walking meditation) and gentle yoga.

Daily homework of 45 minutes meditation was recommended 6 days a week in all 3 trials [24-26].

In all 3 trials, MBSR was taught by 2 instructors each who completed the MBSR teacher training and had a long-standing meditation practice. In 2 trials, 1 of the instructors was a physician [25,26], while in the other trial 1 instructor was an osteopathic physician and the other 1 held a master’s degree in psychotherapy [24].

#### Control conditions

Two RCTs compared MBSR to a waiting list control group [24,25]. Control patients did not receive any specific treatment during the course of the study but were offered the MBSR intervention after the post-treatment assessment. One of the RCTs of older adults [26] compared MBSR to a health education program that controlled for time, group size, and homework. Roughly half of each 90-minute session was dedicated to health-related, mainly back pain-related, education, the other half to mental exercise and discussion. Patients were provided a book and a games console with a "brain training" program as homework.

#### Co-interventions

One RCT explicitly allowed patients in both groups to use additional usual medical care including pain medication during the course of the study [24]. The other 2 RCTs did not specify (dis-)allowance or actual use of co-interventions during the course of the study [25,26].

#### Outcome measures

All 3 RCTs assessed post-intervention pain intensity using visual analog scales (VAS) [24], the McGill Pain questionnaire (MPQ) total score [25,26] or the MPQ current pain score [26]. Disability was also assessed post-intervention by all 3 RCTs, all using the Roland Morris Disability Questionnaire (RMDQ). Two RCTs [24,25] measured pain acceptance post-treatment using the Chronic Pain Acceptance Questionnaire (CPAQ). Two RCTs assessed quality of life [25,26] with the Medical Outcomes Study 36-item short-form survey (SF-36). One trial assessed analgesic use with an analgesic medication log [24] and sleep quality with the Pittsburgh Sleep Quality Index (PSQI) [24]. Another trial assessed self-efficacy using the Chronic Pain Self-Efficacy Scale (CPSS) [26] and mindfulness using the Mindful Attention Awareness Scale (MAAS) and the Five Facet Mindfulness Questionnaire (FFMQ) [26].

Only one RCT [26] reported group comparisons at longer-term follow-up.

### Risk of bias

Risk of bias for each study is shown in Table 2. Risk of selection bias was low in all included RCTs. Only 1 study [26] reported blinding of outcome assessment and no study reported blinding of participants and personnel. However, one study [26] used an adequate active comparison group and treatment expectancy was comparably high in intervention and control group at baseline and post-treatment. Therefore it was judged that outcomes in this study were not likely to be influenced by lack of blinding. Risk of attrition bias was high in 2 out of 3 RCTs, while risk of reporting bias and other bias were low in all 3 RCTs.

**Table 2:** Risk of bias assessment of the included studies using the Cochrane risk of bias tool

**Bias**	**Random sequence generation (selection bias)**	**Allocation concealment (selection bias)**	**Blinding of participants and personnel (performance bias)**	**Blinding of outcome assessment (detection bias)**	**Incomplete outcome data (attrition bias)**	**Selective reporting (reporting bias)**	**Other bias**
**Author, year**							
Esmer et al. 2010 [24]	Low risk^a^	Unclear	High risk	High risk	High risk	Low risk	Low risk
Morone et al. 2008 [25]	Low risk	Low risk	Unclear	Unclear	Low risk	Low risk	Low risk
Morone et al. 2009 [26]	Low risk	Low risk	Low risk	Low risk	High risk	Low risk	Low risk

^a^Additional details provided upon request.

### Effectiveness of MBSR compared to no treatment for chronic low back pain

One trial on mixed non-specific and specific chronic low back pain in older adults did not find any differences between MBSR and a wait-listed control group on pain intensity on the MPQ or back-specific disability as assessed with the RMDQ [25]. While disability improved within the MBSR group, group differences were not of clinical importance. This RCT reported MBSR being superior to wait-list in improving physical functioning, but not bodily pain, global health composite, physical health composite, or mental health composite on the SF-36. Pain acceptance on the CPAQ was reported to be significantly higher after MBSR as compared to no treatment. No differences in outcomes within the MBSR group were reported from end of intervention to 1-month follow-up.

One RCT on failed back surgery syndrome reported significant group differences between MBSR and a wait-listed control group in change of pain intensity immediately after the intervention period [24]. The difference in change scores between groups (MBSR: -6.9 cm vs. wait-list: -0.2 cm; sum score of 3 10 cm-VAS) was deemed clinically important. Significant and clinically important group differences after the intervention also were reported for change in disability on the RMDQ (MBSR: -3.6 vs. wait-list +0.1). Further, larger improvements were found for pain acceptance on the CPAQ, medication intake, and sleep quality on the PSQI for the MBSR group. While no group differences were assessed at 40-week follow-up, improvements in the MBSR group were reported to persist at this time point.

### Effectiveness of MBSR compared to health education for chronic low back pain

One RCT on mixed non-specific and specific chronic low back pain in older adults reported no differences between MBSR and health education on pain intensity on the MPQ or back-specific disability on the RMDQ [26]. While disability improved in both groups, group differences did not reach clinical importance. Group differences at short-term follow-up were reported for emotional role functioning on the SF-36, but not for bodily pain on the SF-36, self-efficacy on the CPSS or mindfulness on the MAAS or the FFMQ [26]. No group differences in disability, pain intensity, self-efficacy, quality of life or mindfulness were found at 4-month follow-up.

### Safety

One RCT did neither report occurrence (or absence) of adverse events nor reasons for drop-outs [24]. Another RCT reported that no serious adverse events occurred [25]. However, 3 patients dropped out from the MBSR group due to unexpected health or family obligations [25]. The third RCT reported that there were no adverse events or drop-outs due to health obligations [26].

## Discussion

This systematic review found only limited evidence that MBSR can provide short-term relief of pain and back-related disability in low back pain patients. Statistical significant and clinically relevant group differences were reported in only 1 out of 3 RCTs. Single studies reported effects on physical or emotional well-being but overall, only little effects on quality of life were reported. These results are only partly in line with a recent meta-analysis on mindfulness-based interventions for chronic pain that found MBSR to be superior to controls in reducing pain intensity and increasing physical wellbeing but not in increasing quality of life [14]. However, this meta-analysis included only 1 of the RCTs included in the present review [25].

Methodological differences between the included RCTs might explain some of the differences in results: firstly, different control groups were chosen; while 1 RCT used an adequate active control group [26], 2 RCTs compared MBSR to no treatment [24,25] and 1 of those was the only study that reported positive intervention effects on most of the study outcomes [24]. Secondly, another source of heterogeneity are differences in inclusion criteria between studies: the study that showed favorable effects of MBSR included a sample of highly chronified specific low back pain patients [24] while the 2 trials that showed little effects included patients with specific or unspecific low back pain [25,26]. Moreover, the 2 RCTs that did not report significant group differences in pain intensity or back-related disability included only older adults [25,26] while no age restriction was posed in the only RCT that reported effectiveness of MBSR for most outcome measures [24]. It has been argued that standard pain measurement instruments might not be suitable for elderly patients [27,28]. Specialized comprehensive approaches might be needed to correctly assess pain intensity in elderly patients [28]. Thirdly, the 2 RCTs that did not report significant group differences did not include yoga or an all-day retreat in their MBSR program [25,26]. Yoga has been reported to increase back-related function and to decrease disability in patients suffering from low back pain [29,30]. As the only RCT that reported favorable effects of MBSR on functional disability actually included yoga in the MBSR program [24], yoga might be crucial for this effect. Further research should include dismantling studies that separately evaluate the effects of different components of MBSR such as mindful meditation and yoga.

Although the use of pain intensity and disability as main outcome measures is in accordance with the IMMPACT recommendations [31], pain relief is not the main aim of MBSR [14]. Instead, patients are guided to accept all varieties of experience, be them pleasant or unpleasant, without elaboration or judgment [5,6]. In accordance with this approach, 2 RCTs reported increased pain acceptance after MBSR interventions [24,25]. Pain acceptance describes patients’ attempt to maintain function in spite of their pain as far as possible [32]. Higher pain acceptance has been found to be associated with lower pain intensity and disability [33]. However, whether or not pain acceptance is a mechanism by which MBSR relieves pain in low back pain patients is beyond the scope of this review.

At the moment there is no evidence for longer-term effects of MBSR in low back pain. More RCTs with longer follow-ups are needed.

Generally, adverse events and reasons for drop-outs were poorly reported. This is unsatisfying since safety is a major issue in evaluating therapies. Further trials should put a focus on detailed reporting of safety data.

All included RCTs used MBSR as an intervention. No RCT assessing the effectiveness of MBCT in low back pain patients could be located. This is in line with the aforementioned meta-analysis of chronic pain that could not locate any trials on MBCT either [14].

The evidence found in this review is clearly limited due to several reasons. Firstly, the total number of eligible RCTs was small and clinical heterogeneity was high between RCTs. Thus, no meta-analysis could be performed. This review only included trials that were published in peer reviewed scientific journals. Therefore, some RCTs that were published in “grey literature” or conference proceedings only might have been missed. Secondly, the total number of included patients was low. No study included more than 20 patients in each group. More large RCTs are needed to definitely judge the effects of MBSR in low back pain. Thirdly, the evidence was suspect to high attrition bias. Fourthly, 2 out of 3 RCTs compared MBSR with wait-lists. While there is limited evidence that MBSR is effective in low back pain, more research is needed to evaluate superiority or inferiority of MBSR to other active treatments.

## Conclusions

This systematic review found only inconclusive evidence of short-term effectiveness of MBSR in improving pain intensity and disability in patients suffering from low back pain. However, there is limited evidence from 2 wait-list controlled trials that MBSR can improve pain acceptance. Further trials with larger sample size, active control groups and longer follow-up are needed before the evidence for MBSR in low back pain can conclusively be judged.

## Competing interests

All authors disclose any commercial association that might create a conflict of interest in connection with the submitted manuscript. There is especially no competing financial interest for any of the authors.

## Authors’ contributions

HC was responsible for conception and design of the review, carried out the literature search, performed data analysis, and drafted the manuscript. HH and RL performed data extraction and assessment of risk of bias, participated in conception and design of the review, and critically revised the manuscript. GD participated in conception and design of the review, and critically revised the manuscript. All authors read and approved the final manuscript.

## Pre-publication history

The pre-publication history for this paper can be accessed here:

http://www.biomedcentral.com/1472-6882/12/162/prepub
